# Supplementary material for: Maternal environment alters dead pericarp biochemical properties of the desert annual plant Anastatica hierochuntica L
Source: PLoS One. 2020 Jul 31;15(7):e0237045. doi: 10.1371/journal.pone.0237045 (PMC7394380; doi:10.1371/journal.pone.0237045)
Supplement: S1 File — (PDF) [file pone.0237045.s001.pdf]

Supplemental materials

Maternal environment alters dead pericarp biochemical properties of the desert annual plant *Anastatica hierochuntica* L.

Janardan Khadka†[1], Buzi Raviv†[1], Bupur Swetha[1], Rohith Grandhi[1], Jeevan R. Singiri[1], Nurit Novoplansky[1], Yitzchak Gutterman[1], Ivan Galis[2], Zhenying Huang[3], Gideon Grafi[1]*

[1]French Associates Institute for Agriculture and Biotechnology of Drylands, Jacob Blaustein Institutes for Desert Research, Ben-Gurion University of the Negev, Midreshet Ben Gurion 84990, Israel. [2]Institute of Plant Science and Resources, Okayama University, Kurashiki, Okayama, 710-0046, Japan. [3]State Key Laboratory of Vegetation and Environmental Change, Institute of Botany, Chinese Academy of Sciences, Beijing, China.

†Equal contribution

*Corresponding author: Gideon Grafi, email: ggrafi@bgu.ac.il

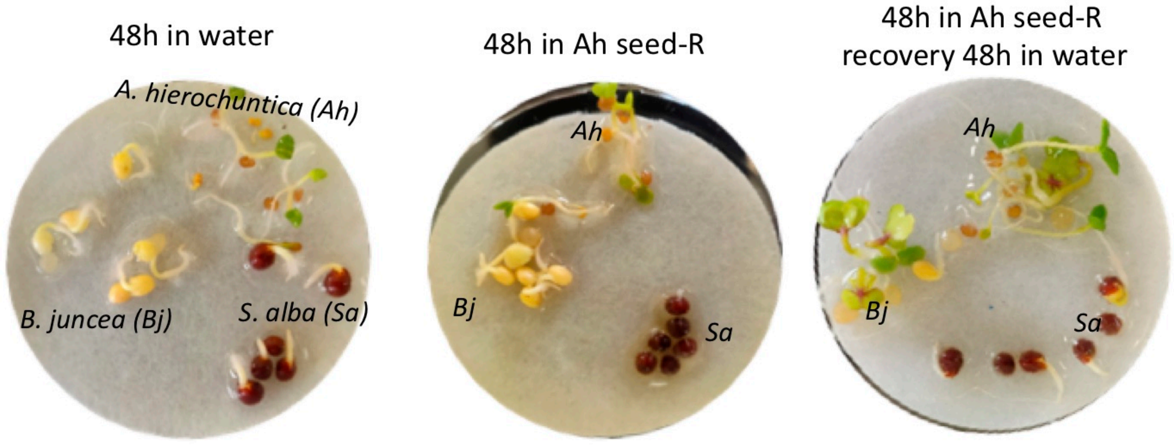

Fig. S1. Seed secretion of *A. hierochuntica* contains species-specific germination inhibitory substances. Seeds of *A. hierochuntica (Ah)*, *Brassica juncea (Bj)* and *Sinapis alba (Sa)* were germinated for 48 h in water or in extract containing substances released from *Anastatica* seeds (Ah seed-R) followed by extensive washing with water and recovery for 48 h.

A

B

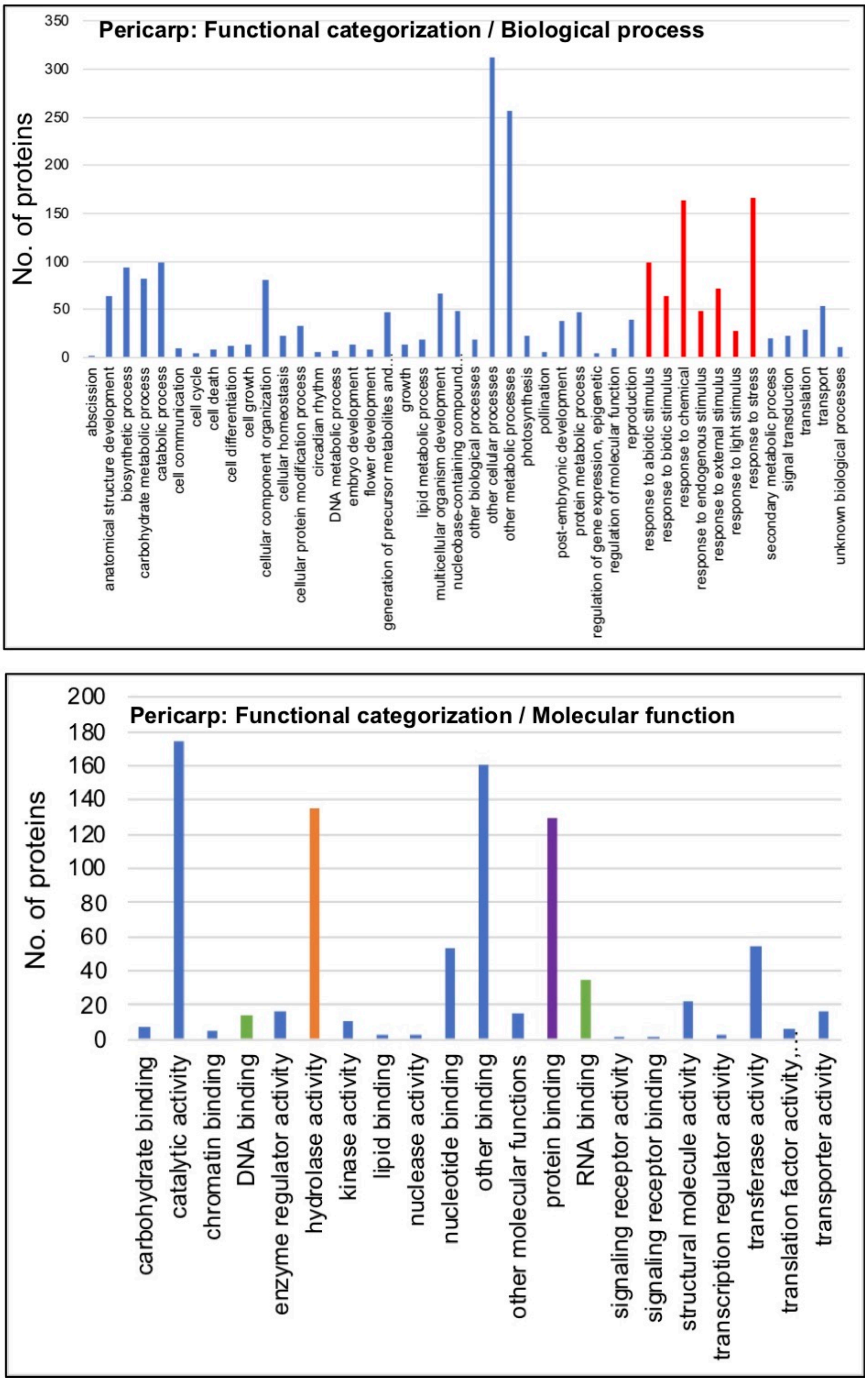

Fig. S2. GO categorization of proteins extracted from *A. hierochuntica* dead pericarps. A. Biological process - Proteins categories involved in response to various stimuli are highlighted red. B. Molecular function – Highly represented classes of hydrolase, RNA and DNA binding and protein binding are highlighted orange, green and purple, respectively. Functional categorization was performed using Gene Ontology in TAIR [1].

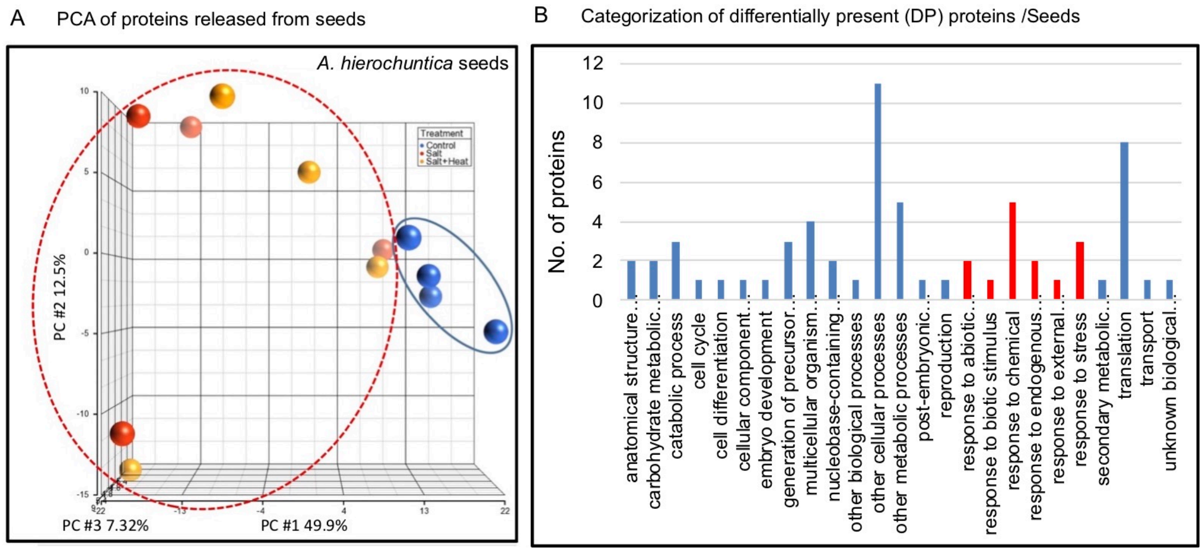

Fig. S3. Proteome analysis. (A) Principal component analysis (PCA) score plots comparing the proteome profiles of proteins released upon hydration from seeds obtained from mother plants of *A. hierochuntica* exposed to stress conditions (red and yellow dots) and control, untreated plants (blue dots). **B**. Biological process categorization of differentially present (DP) proteins released from seeds derived from control and stress-treated plants. Categories related to response to various stimuli are highlighted red.

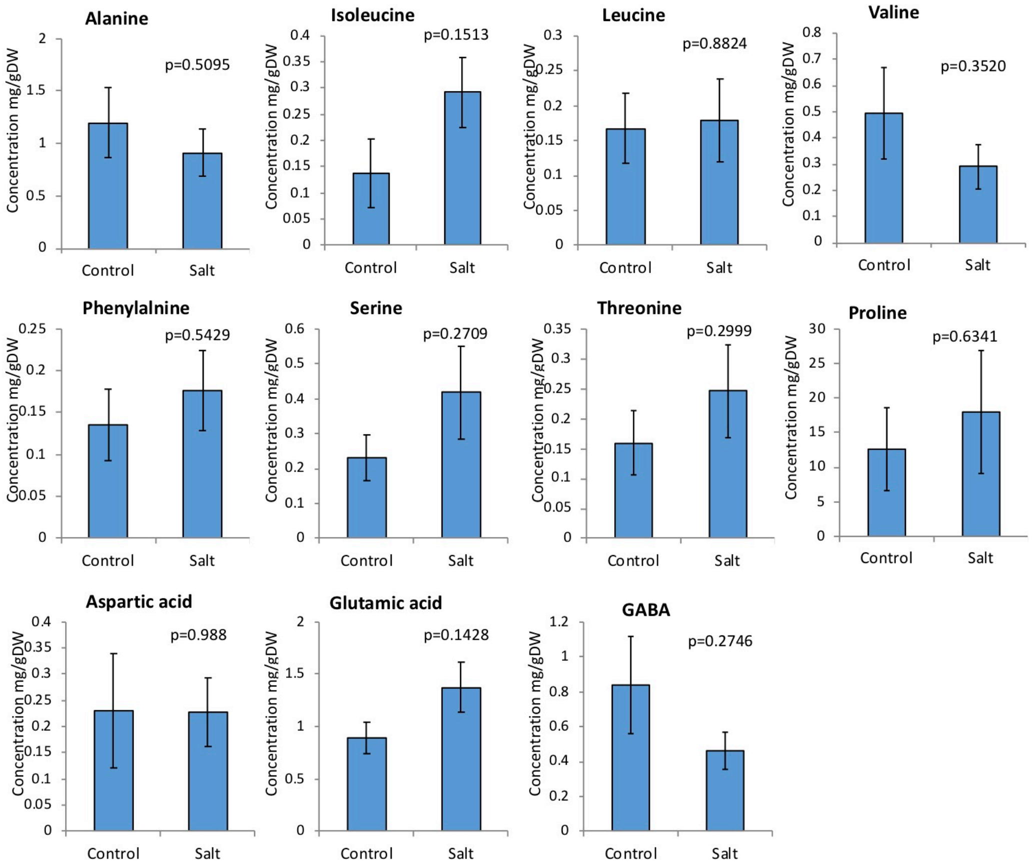

Fig. S4. GCMS analysis of the indicated amino acids recovered from *Anastatica* pericarps derived from plants grown under control and salt conditions. Vertical bar is the standard error (n=4). GABA is γ-aminobutyric acid. p-values below 0.05 are considered statistically significant.

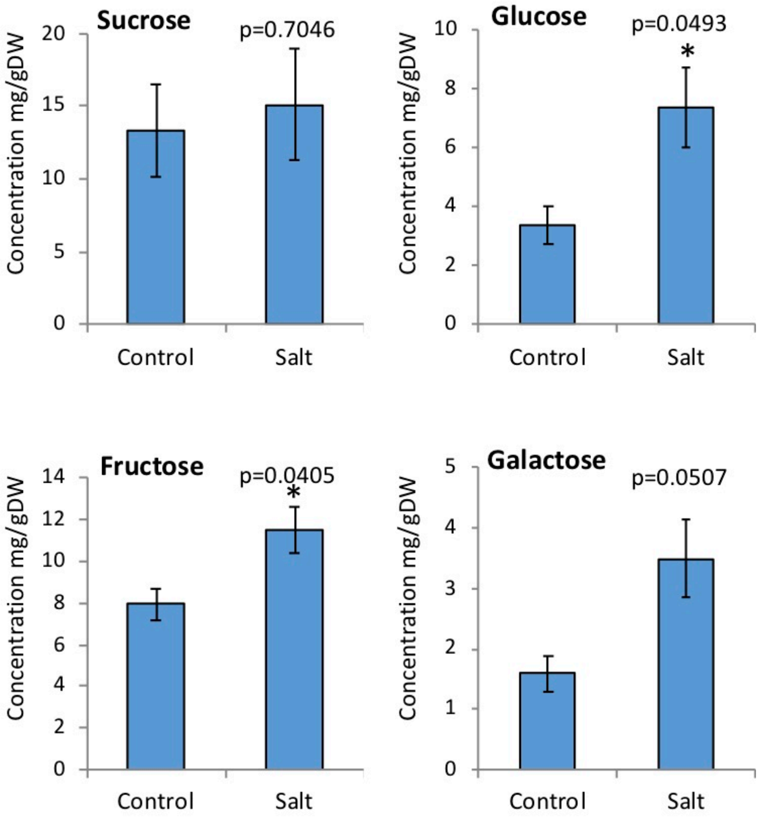

Fig. S5. GCMS analysis of the indicated sugars recovered from *Anastatica* pericarps derived from plants grown under control and salt conditions. Vertical bar is the standard error (n=4). p-values below 0.05 are considered statistically significant.

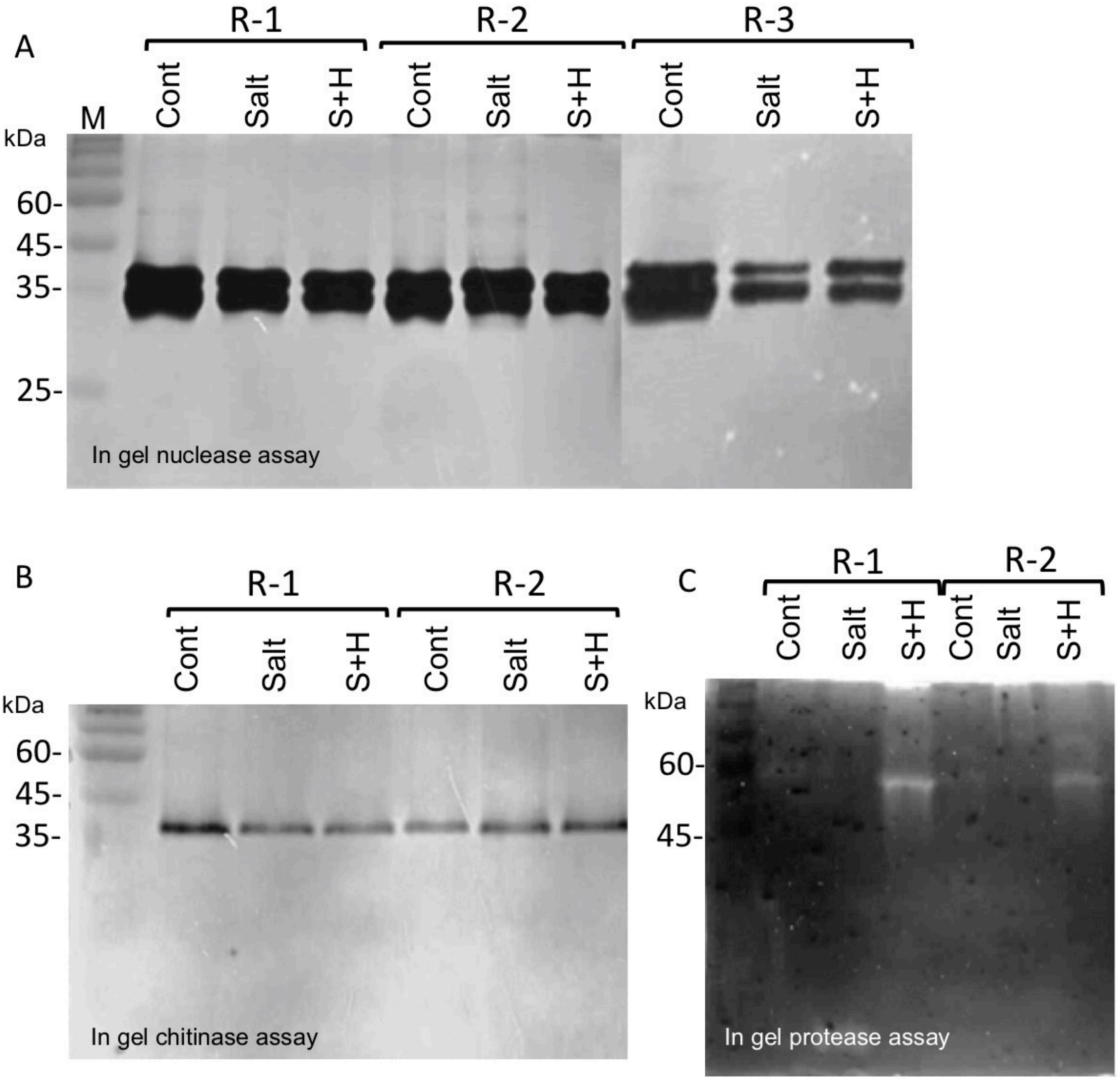

S6 Fig. The essentially original uncropped and unadjusted images underlying gel results presented in Fig 4 of the manuscript.

Analysis of hydrolase activities in pericarps derived from stress-treated plants by in gel assays. In gel nucleas assay (A), chitinase assay (B) and protease assay (C) were performed on proteins released from pericarps derived from control (Cont) plants or plants treated with salt or with a combination of salt and heat (S+H). Pericarps from two separate experiments R-1 and R-2 were analyzed. R-3 in A refers to experiments were all replicas were combined and analyzed with reduced extract concentration. M, protein molecular weight markers.

References

1. Berardini TZ, Mundodi S, Reiser R, Huala E, Garcia-Hernandez M, Zhang P, Mueller LM, Yoon J, Doyle A, Lander G, Moseyko N, Yoo D, Xu I, Zoeckler B, Montoya M, Miller N, Weems D, Rhee SY. Functional annotation of the Arabidopsis genome using controlled vocabularies. Plant Physiol. 2004; 135: 1-11.
